# Hydrological impacts of precipitation extremes in the Huaihe River Basin, China

**DOI:** 10.1186/s40064-016-3429-1

**Published:** 2016-10-06

**Authors:** Mangen Yang, Xing Chen, Chad Shouquan Cheng

**Affiliations:** 1 School of Atmospheric Sciences, Nanjing University, Nanjing, 210023 Jiangsu China; 2 CMA-NJU Joint Laboratory for Climate Prediction Studies, Nanjing University, Nanjing, 210023 Jiangsu China; 3 School of Geographical Sciences, Fujian Normal University, Fuzhou, 350007 Fujian China; 4 Science Section, Operations—Ontario, Meteorological Service of Canada, Environment Canada, Toronto, Canada

**Keywords:** Precipitation indices, Precipitation extremes, Hydrological impacts, Streamflow, Huaihe River Basin, China

## Abstract

Precipitation extremes play a key role in flooding risks over the Huaihe River Basin, which is important to understand their hydrological impacts. Based on observed daily precipitation and streamflow data from 1958 to 2009, eight precipitation indices and three streamflow indices were calculated for the study of hydrological impacts of precipitation extremes. The results indicate that the wet condition intensified in the summer wet season and the drought condition was getting worse in the autumn dry season in the later years of the past 50 years. The river basin had experienced higher heavy rainfall-related flooding risks in summer and more severe drought in autumn in the later of the period. The extreme precipitation events or consecutive heavy rain day events led to the substantial increases in streamflow extremes, which are the main causes of frequent floods in the Huaihe River Basin. The large inter-annual variation of precipitation anomalies in the upper and central Huaihe River Basin are the major contributor for the regional frequent floods and droughts.

## Background

Meteorological extreme events have been paid more and more attention from all levels of the governments and communities because of their more frequent occurring and more devastating impacts on infrastructures and human daily life over the globe. Third assessment report of the intergovernmental panel on climate change (IPCC) pointed out that the extremes referred to the rare events based on a statistical model of particular weather elements, and changes in the extreme events may relate to changes in the mean and variance (IPCC 2001). Furthermore, IPCC fourth assessment report summarized the characteristics of precipitation extremes at the global and regional scales and indicated that the frequency of heavy precipitation events increased over most land areas (IPCC 2007). Recently, IPCC Fifth Assessment Report also indicated that the number of heavy precipitation events over land has increased in more regions since the mid-20th century and floods larger than recorded since the 20th century occurred during the past five centuries in eastern Asia (IPCC 2013). The research on observed precipitation revealed a distinct link between rainfall extremes and temperature, with the increasing in heavy rainfall events during the warm periods and the decreasing during the cold periods (Allan and Soden 2008). Furthermore, much research indicated that extreme precipitation events were very sensitive to global climate change, so a small change in average climate may cause large changes in the statistics of precipitation extreme events (Groisman et al.^1999^; Easterling et al. 2000; Meehl et al. 2000; Groisman et al. 2005).

At the global scale, precipitation changes showed a widespread and significant increase, but the changes were much less spatially coherent compared with temperature changes (Alexander et al. 2006). At the regional scale, many studies also showed that there were less spatial or temporal coherence in precipitation changes. Based on various precipitation indices, these studies focused on various specific regions, such as Southeast Asia and the South Pacific (Manton et al. 2001), Eastern Mediterranean (Kostopoulou and Jones 2005), Western Indian Ocean (Vincent et al. 2011), southern Poland and central-eastern Germany (Lupikasza et al. 2011), northwest Mexico and southwest United States (Sarahí and Cavazos 2010), Central and Western Europe(Moberg and Jones 2005), South Africa (Kruger 2006), Central America and northern South America (Aguilar et al. 2005), and Asia–Pacific Network region (Choi et al. 2009). On the other hand, many other studies focused on specific nation or locality, such as Greece (Kioutsioukis et al. 2010), Bulgaria (Bocheva et al. 2009), India (Roy and Balling 2004, 2009), and the US (Karl and Knight 1998; Kunkel et al. 1999; Michael and Bradley 2007; Pal and Al-Tabbaa 2009; Brown et al. 2010; Chu et al. 2010; Mishra and Singh 2010; Santos et al. 2011). Therefore, the studies on precipitation changes at the regional scale or local scale are of important practical value because of their great spatial variations.

In China, the frequency change of precipitation extremes showed a large spatial variation (Zhai et al. 1999). In the Yangtze River basin and the southeast coastal area of China, the frequency of extreme precipitation events showed upward trends (Su et al. 2006; Ren 2007; Zhang et al. 2007; Ding 2008), while in the northern China there were downward trends (Zhang et al. 2008) during the historical observation period. In the Tibetan Plateau, the number of the heavy-rain days had non-significant upward trends, while maximum 5-day precipitation, consecutive dry days and consecutive wet days showed downward trends (You et al. 2008). Over the Circum-Bohai-Sea region, there were significant decreasing in summer precipitation, frequency and intensity of extreme precipitation events (Jiang et al. 2011).

Precipitation is one of the most important elements of the hydrological cycle. Disasters associated with heavy precipitation, such as floods, landslides, and mud-rock flows, affect directly on the natural ecological and social economic systems. However, only a few studies focused on precipitation extremes on the river-basin scale (Hundecha and Bárdossy 2005; Bartholy and Pongrácz 2007, 2010; Cheng et al. 2010, 2011; Gemmer et al. 2011; Wang et al. 2008; Zhan et al. 2011). Most of these studies focused on the characteristics of precipitation extremes, and only a few studies investigated the relationship between the precipitation extremes and the high streamflow events (Dong et al. 2011; Wang et al. 2008).

This study will focus on the hydrological impacts of precipitation extremes in the Huaihe River Basin. A major hydro-meteorological feature in the Huaihe River Basin is frequent occurrence of floods and droughts. Some previous studies suggested that the upper reach of the Huaihe River Basin had the highest probability of extreme rainfall events (e.g. Dong et al. 2011) and the increased rainfall had great impacts on runoff (Zhang et al. 2009). Within the Huaihe River Basin, the precipitation-runoff responses are different in the different sub-basins, which are reflected by a variety of physical geographical characteristics (Wang et al. 2003). Therefore, the major purpose of this study is to analyze the hydrological impacts of spatial–temporal patterns of precipitation extremes in the Huaihe River Basin.

This paper is organized as follows. “Materials and methods” section describes materials and methods applied to the Huaihe River basin, data sources and treatment, and analysis methods. “Results and discussion” section summarizes the results and discussion on the characteristics of precipitation extremes and their hydrological impacts. The conclusions from the study are presented in “Conclusions” section.

## Materials and methods

### Huaihe River Basin

**Fig. 1:**
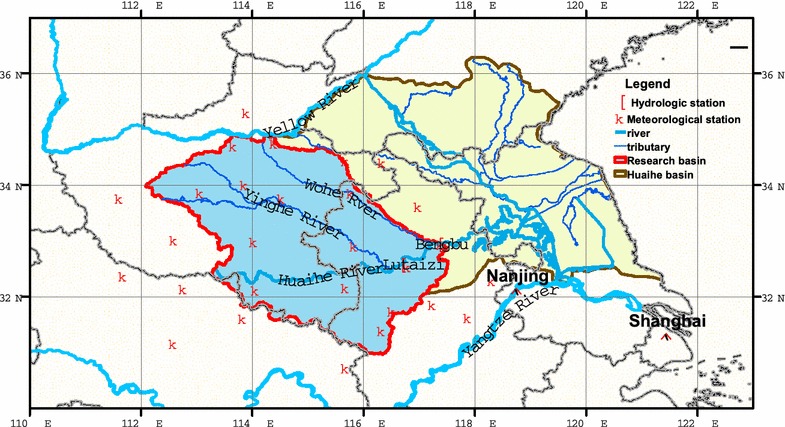
The study area and monitoring stations in the Huaihe River Basin

The Huaihe River Basin is located in the eastern China from 115°E to 118.5°E and from 30.5°N to 35.5°N (shown in Fig. 1), covering an area about 2.70 × 10^5^ km^2^ between the Yangtze River and the Yellow River. The basin is situated in the East Asian monsoon climate region, in the climate transition zone from the humid southern region to the semi-humid northern region. Mean annual temperature ranges from 11 °C to 16 °C, with the highest monthly mean temperature (25 °C) in July and the lowest monthly mean temperature (0 °C) in January. Basin-averaged annual precipitation is 883 mm calculated from 28 meteorological stations, ranging from 600 to 1400 mm. Precipitation in the basin is mainly controlled by the summer monsoon system, and heavy rainfall events usually occur in the rainy season from May to September with high inter-annual rainfall variability.

Hydrological station Bengbu is located at the south bank of the Huaihe River in the province of Anhui, which is an important hydrological gauging station on the Huaihe River mainstream (Fig. 1). As the main runoff-generating area of whole Huaihe River Basin, the study area, two-third of the basin is covered by plains (lakes, depressions), and others are low mountains, hills, and highlands. (The elevation of the basin ranges from 24 to 1684 m, with ninety percent of the basin area below 300 m. There are two long tributaries, the Wohe River and the Yinghe River meandering over the northern basin as shown in Fig. 1. Main tributaries of the Huaihe River are from the upper-basin mountainous and highland area down to the lower-basin mainstream at the same node, which geographically makes the Huaihe River Basin be one of the basins with the highest flooding risks in China.

### Data sources and treatment

Observed daily precipitation data for 45 meteorological stations in the study basin is from the National Climate Center of China (NCCC), China Meteorological Administration (CMA). Karl and Knight (1998) pointed out that there were some errors in precipitation trend analysis if missing data with time dependence were replaced by zero or monthly mean values. In light of this concern, the meteorological stations in the basin were selected based on the length and completeness of precipitation record. If there are more than 10 % days data missing in a year, that year is considered as a data-missing year (Zhai et al. 2005). When the data-missing years at a station are more than 5 years, the station is excluded from the study (Tank and Können 2003). Based on the analysis, 28 meteorological stations are qualified within the basin, as shown in Fig. 1.

Daily streamflow data at the hydrological station Bengbu for time period 1958–2009 were used in this study. Daily streamflow data at the station is missing in the periods January–April 1977 and September–December 1978. The missing data in these two periods were estimated by using streamflow data at the hydrological station Lutaizi, which is located at upstream of Bengbu (Fig. 1). Considering hydrological connections of the streamflow between the two stations, we employed regression analysis for two streamflow datasets with 1-day, 2-day, and 3-day lags. The results showed that there is a strong correlation in 1-day lag between two streamflow datasets with the model R^2^ = 0.93. Then the streamflow missing data at the hydrological station Bengbu were interpolated by the regression equation, making it possible to analyze the hydrological impacts of precipitation from 1958 to 2009.

### Methods

Climate extremes can be placed into two broad groups: (1) those based on simple climate statistics, which include extremes such as a very low or very high daily temperature, or daily or monthly heavy rainfall amount, that occur every year; and (2) more complex event-driven extremes, include drought, flood, or hurricanes, which may not occur every year at a specific location. The detection of changes in extremes on the basis of climate statistics is much more likely than the detection of event-driven extremes (Easterling et al. 2000). Based on climate statistics, the expert team on climate change detection, monitoring and indices (ETCCDMI) of climate variability and predictability (CLIVAR) project defined a set of indices for temperature and precipitation extremes to gain insight to the changes in extremes. Twenty-seven indices were defined based on daily temperature values (minimum, maximum) or daily precipitation amounts, including eleven precipitation indices (Nicholls and Murray 1999). For these precipitation indices, some were calculated on the basis of station-related thresholds, while others were based on fixed thresholds or absolute peak values. Since the Huaihe River Basin is located in the climate transitional zone and its precipitation climatology shows great difference between the south and the north, the precipitation indices based on fixed thresholds or absolute peak values are not appropriate. Among the eleven precipitation indices, seven station-related precipitation indices were selected to investigate the characteristics of precipitation extremes in the Huaihe River Basin (Table 1). In addition to these seven indices, another index—precipitation probability (PRCPprb) was introduced in the analysis. To analyze the trends of time series and seasonal variations of extreme precipitation and their impacts on streamflow, these indices were calculated on both annual and monthly bases. All eight precipitation indices on annual basis were employed for the trend analysis; four of them (PRCPtot, PRCPprb, RX1 day, RX5 day) on monthly basis were applied to seasonal variation analysis.

**Table 1:** Precipitation indices used in the study

Index	Explanation	Definition	Unit
CDD	Consecutive dry days	Maximum number of consecutive days with daily precipitation <1 mm	day
CWD	Consecutive wet days	Maximum number of consecutive days with daily precipitation ≥1 mm	day
PRCPprb	Precipitation probability	Probability of wet days (with precipitation ≥1 mm)	%
PRCPtot	Annual total wet-day precipitation	Annual or monthly total precipitation in wet days (precipitation ≥1 mm)	mm
R95p	Heavy precipitation totals	Annual total precipitation when precipitation >95th percentile	mm
RX1 day	Max 1-day precipitation amount	Annual or monthly maximum 1-day precipitation amount	mm
RX5 day	Max 5-day precipitation amount	Annual or monthly maximum precipitation amount in 5 consecutive days	mm
SDII	Simple precipitation intensity indicator	Total annual precipitation divided by the total number of wet days (with precipitation ≥1 mm)	mm/day
MinFLow	Minimum streamflow	Annual or monthly minimum streamflow	m^3^/s
FLow	Mean streamflow	Annual or monthly mean streamflow	m^3^/s
FX1 day	Maximum 1-day streamflow	Annual or monthly maximum 1-day streamflow	m^3^/s
FX5 day	Maximum 5-day mean streamflow	Annual or monthly maximum streamflow in 5 consecutive days	m^3^/s

According to the definition of RX1 day and RX5 day, two streamflow indices, maximum 1-day mean streamflow (FX1 day) and maximum 5-day mean streamflow (FX5 day) were calculated on both annual and monthly bases from daily streamflow data at the hydrological station Bengbu for the time period 1958–2009. Other two streamflow indices, minimum streamflow (MinFLow) and mean streamflow (FLow) were also calculated on both annual and monthly bases.

In this study, Sen’s slope estimator (Sen 1968) was used to analyze trends of time series for precipitation indices and streamflow indices. Non-parametric Mann–Kendall test was used to statistically determine the significance level of the trends (Mann 1945; Kendall 1975). Mann–Kendall test was selected for the analysis because the statistic was based on sign of differences but not directly on values of the random variable; consequently, trends determined were less affected by outliers (Mishra and Singh 2010). Similarly, Sen’s slope method was also not greatly affected by single data value or outlier (Chu et al. 2010).

Correlation is a term that refers to the strength of a relationship between two variables and correlation analysis is one of the most widely used in scientific research. There are several types of correlation coefficients, such as Pearson’s and Spearman’s rho, are the most commonly used. In this study, Pearson’s correlation coefficient was used to represent the strength of the relationship between precipitation indices and streamflow indices.

Since the study basin has flat terrain and the meteorological stations are well-distributed, each precipitation index arithmetically averaged from 28 stations, representing their basin-averaged value (Nicholls and Murray 1999). In order to find the appropriate spatial interpolation method for each precipitation index, the following experiment scheme was designed. Among the 28 meteorological stations, twenty of them were used to spatially interpolate the indices and the remaining eight stations were used to verify the interpolated results. The spatial interpolation experiment was run four times with updating four new stations to replace half of eight verification stations at each time. The interpolation experiment was carried out in Arc Geographical Information System (ArcGIS) and three interpolation methods, the inverse distance weighting (IDW), Ordinary Kriging method (Kriging) and Spline method (Spline) were tested. The mean square error (MSE) was used as a criterion to evaluate and select the spatial interpolation methods. The evaluation results and the best selected spatial interpolation method for each index are listed in Table 2.

**Table 2:** Selected spatial interpolation methods for precipitation indices based on the mean square error (MSE) evaluation results (*IDW* inverse distance weighting)

Index	MSE	CDD	CWD	PRCPprb	PRCPtot	R95p	RX1 day	RX5 day	SDII
Value	Method	Kriging	Kriging	Kriging	Kriging	Kriging	Kriging	Kriging	Kriging
	MSE	1.58	0.21	0.0067	41.98	15.66	5.67	7.70	0.37
	Method	IDW	IDW	IDW	IDW	IDW	IDW	IDW	IDW
	MSE	2.06	0.25	0.0093	39.88	16.43	4.76	7.87	0.45
	Method	Spline	Spline	Spline	Spline	Spline	Spline	Spline	Spline
	MSE	2.52	0.27	0.0118	51.80	14.66	4.66	5.78	0.33
	Selected methods	Kriging	Kriging	Kriging	IDW	Spline	Spline	Spline	Spline
Anomaly	Method	Kriging	Kriging	Kriging	Kriging	Kriging	Kriging	Kriging	Kriging
	MSE	2.53	0.40	0.0041	47.80	33.84	11.87	23.80	0.52
	Method	IDW	IDW	IDW	IDW	IDW	IDW	IDW	IDW
	MSE	1.87	0.37	0.0046	46.79	38.92	9.57	25.47	0.50
	Method	Spline	Spline	Spline	Spline	Spline	Spline	Spline	Spline
	MSE	1.83	0.27	0.0077	38.79	33.12	9.03	23.17	0.48
	Selected methods	Spline	Spline	Kriging	Spline	Spline	Spline	Spline	Spline

## Results and discussion

### Characteristics of precipitation and streamflow extremes

The trends of indices for basin-averaged precipitation and streamflow indices are presented in Table 3. All indices show upward trends except for the consecutive dry days (CDD) and precipitation probability (PRCPprb). Among the precipitation indices, only downward trend of CDD and upward trend of RX1dayare statistically significant (α = 0.05, similarly hereinafter). During the study period 1958–2009, the basin-averaged CDD decreased by 3.06 days per decade, while RX1 day increased by 2.34 mm per decade. In general, annual CDD events occur in dry season while annual RX1 day events occur in wet season. This suggests that the drought condition was getting worse in dry season, precipitation intensified in wet season and seasonal contrast of precipitation increased in the later years of the past 50 years. All streamflow indices show weak upward trends and are not statistically significant.

**Table 3:** Annual time series trend slopes of basin-averaged precipitation indices and streamflow indices in Bengbu Station during the period 1958–2009 (per decade)

CDD	CWD	PRCPprb	PRCPtot	R95p	RX1 day	RX5 day	SDII	FLow	FX1 day	FX5 day
−*3.06*	0.02	-0.002	15.70	13.98	*2.34*	2.73	0. 22	0.42	167.13	161.31

Italic denotes the trends are statistically significant [α = 0.05] and the rest non-significant

To investigate seasonal differences in the trends of precipitation and streamflow extremes, the monthly-based precipitation and streamflow indices were compared for each month. As shown in Table 4, the trends of four precipitation indices are statistically significant in February (PRCPprb, PRCPtot, RX1 day, RX5 day), September (PRCPprb, RX1 day, RX5 day), July (RX1 day, RX5 day), and January (PRCPprb, RX5 day), respectively; but the trends of all streamflow indices are not statistically significant. All precipitation indices possessed positive Sen’s slope estimator in summer (June, July and August) and negative Sen’s slope estimator in autumn (September, October and November). These results imply that the seasonal contrast of precipitation between summer (wet) and autumn (dry) became more significant in the basin during the past 50 years. In some months, the streamflow and precipitation indices have the same trends, such as upward trend in February, July and August, and downward trend in October and November. However, in some other months, the trends of the streamflow and precipitation indices are opposite, such as in September, May and December. Except precipitation, there must be other factors, such as human activities and land surface cover, affecting or controlling the streamflow (IPCC 2001). More than 3000 reservoirs have been constructed in the Huaihe River Basin since 1951, with total reservoir capacity 20.2 billion m^3^ (Ren 2011). Furthermore, agricultural water demand in September reaches generally the maximum throughout a year (Xu and Ou 2012). Generally speaking, during the past 50 years, the seasonal contrast of precipitation increased, but the seasonal variation of streamflow was greatly influenced by human activities, such as reservoirs regulating and agricultural water demand.

**Table 4:** Monthly time series trend slopes of the precipitation indices and streamflow indices during the period 1958–2009 (per decade)

Index	Jan	Feb	Mar	Apr	May	Jun	Jul	Aug	Sep	Oct	Nov	Dec
PRCPprb	*0.02*	*0.03*	−0.01	**-** *0.03*	-0.01	0.00	0.00	0.01	−*0. 02*	−0.01	−0. 01	0. 01
PRCPtot	2.70	*3.56*	0.38	−3.41	1.22	7.05	8.30	5.88	−8.16	−2.86	−0.32	1.87
RX1 day	0.84	*1.06*	−0.07	0.08	0.90	2.30	*3.29*	1.56	−*1.65*	−0.83	−0.42	0.72
RX5 day	*1.75*	*1.59*	0.66	−1.22	0.03	2.26	*5.66*	2.25	−*1.13*	−3.22	−0.59	1.17
FLow	−0.03	0.37	26.88	−20.64	−61.31	−21.95	130.56	81.77	25.72	−36.10	−21.86	−4.06
FX1 day	18.45	7.23	46.26	−60.26	−98.09	70.40	312.92	168.10	125.36	−55.60	−96.15	−9.89
FX5 day	6.47	1.46	44.70	−55.57	−86.86	18.65	288.48	135.84	115.50	−51.80	−59.67	−16.92

Italic denotes the trends are statistically significant [α = 0.05] and the rest non-significant

**Fig. 2:**
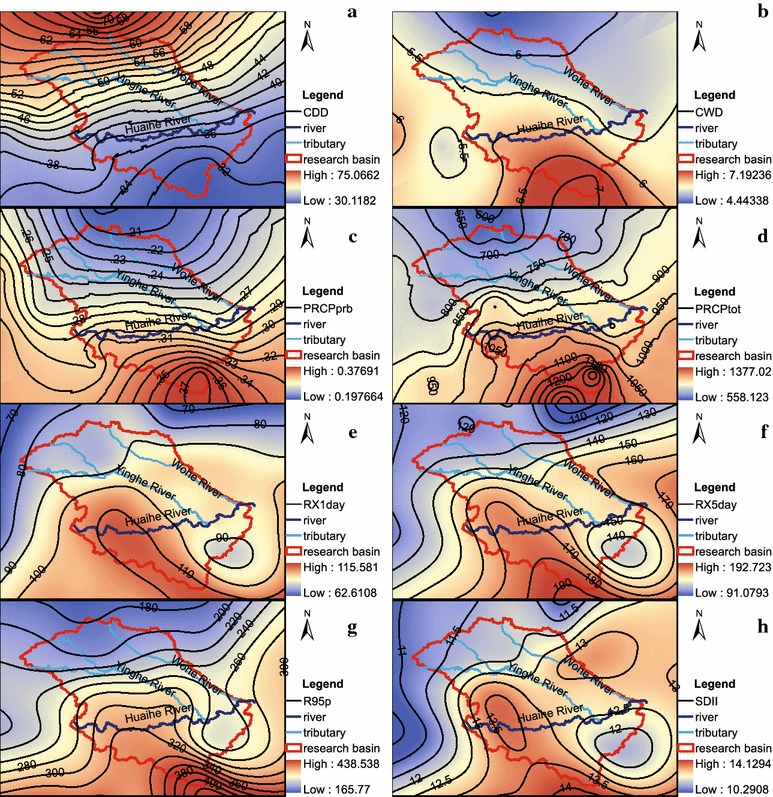
Spatial patterns of the precipitation indices during the period 1958–2009. **a** CDD; **b** CWD; **c** PRCPprb; **d** PRCPtot; **e** RX1day; **f** RX5day; **g** R95p; **h** SDII

In addition to trend analyses of precipitation indices, the spatial distributions of the precipitation indices are presented in Fig. 2. The spatial pattern of CDD shows low values in the south of the Huaihe River Basin and high values in the north (Fig. 2a) with the contours roughly parallel to the river mainstream. Conversely, the spatial pattern of PRCPprb is just opposite to that of CDD, with low values in the north and high values in the south (Fig. 2c). Spatial patterns of CWD and PRCPtot are similar, with high values in the south and the west (upstream) of the basin and low values in the north (Fig. 2b, d). Precipitation intensity indices, such as R95p, RX1 day, RX5 day and SDII, have similar spatial patterns, with differences not only between the south and the north but also between the east and the west. In summary, the low values of the precipitation intensity indices occur in the upper basin of the Wohe River, the Yinghe River, and the Huaihe River; the corresponding high values appear in the south and southeast of the study basin (Fig. 2e–h).

### Hydrological impacts of precipitation extremes

To investigate hydrological impacts of precipitation extremes, we analyzed the relationships between precipitation and streamflow extremes on both annual and monthly bases. Here, the high-streamflow years and low-streamflow years were selected. In addition, the year of 1991 was selected as an example to illustrate the hydrological impacts of precipitation extremes.

The relationships between annual streamflow and precipitation indices are presented in Table 5. It is apparent from Table 5 that the correlations between the streamflow indices (i.e., Flow, FX1 day, FX5 day) and precipitation indices are statistically significant with a few exemptions. These streamflow indices usually have very strong relationships with the precipitation indices, such as PRCPtot, R95p, RX5 day, and RX1 day, the correlation coefficients ranging from 0.70 to 0.87. However, the corresponding relationships between the streamflow indices and PRCPprb are much weaker. And the correlations between CDD and the streamflow indices (i.e., Flow, FX1 day, and FX5 day) are not significant statistically, so that CDD will not be used in the following analysis. These results imply that the indices of heavy rainfall events are more important for streamflow extremes while precipitation probability is less important. The relationships between monthly streamflow indices and monthly precipitation indices are shown in Table 6. All monthly correlations between the precipitation indices and the streamflow indices are statistically significant except December and January due to snowfall effects. In summer season, especially in July t and August, all corresponding correlation coefficients reach their peaks. Therefore, the top risks of heavy rainfall-related flooding in the Huaihe River Basin occur in summer season. The seasonal variation of the precipitation indices between the high- and low-streamflow years further confirms this point (see “Results and discussion” section below).

**Table 5:** Correlation coefficients between basin-averaged precipitation indices and streamflow indices in Bengbu station for the period 1958–2009

Streamflow	CDD	CWD	PRCPprb	PRCPtot	R95p	RX1 day	RX5 day	SDII
MinFLow	−0.20	0.24	*0.36*	*0.33*	0.13	−0.01	0.12	0.14
FLow	−0.12	*0.60*	*0.67*	*0.87*	*0.82*	*0.70*	*0.80*	*0.66*
FX1 day	−0.14	*0.50*	*0.45*	*0.80*	*0.83*	*0.74*	*0.87*	*0.73*
FX5 day	−0.12	*0.50*	*0.45*	*0.79*	*0.83*	*0.74*	*0.87*	*0.73*

Italic denotes the correlations are statistically significant [α = 0.05] and the rest non-significant

**Table 6:** Correlation coefficients in monthly streamflow and monthly precipitation indices during the period 1958–2009

Correlations		Jan	Feb	Mar	Apr	May	Jun	Jul	Aug	Sep	Oct	Nov	Dec
FLow	PRCPprb	0.17	*0.34*	*0.29*	*0.43*	*0.56*	*0.50*	*0.47*	*0.65*	*0.46*	*0.34*	*0.30*	−0.07
	PRCPtot	0.26	*0.35*	*0.46*	*0.54*	*0.58*	*0.49*	*0.66*	*0.75*	*0.43*	*0.47*	0.26	0.03
	RX1 day	0.23	0.25	*0.43*	*0.39*	*0.54*	*0.39*	*0.59*	*0.79*	*0.38*	*0.34*	*0.27*	0.02
	RX5 day	0.23	*0.28*	*0.56*	*0.60*	*0.62*	*0.48*	*0.70*	*0.79*	*0.50*	*0.54*	*0.35*	0.23
FX1 day	PRCPprb	0.18	*0.46*	*0.36*	*0.41*	*0.61*	*0.52*	*0.50*	*0.57*	*0.42*	*0.29*	*0.37*	−0.05
	PRCPtot	0.27	*0.54*	*0.55*	*0.60*	*0.66*	*0.66*	*0.73*	*0.68*	*0.37*	*0.41*	*0.31*	0.03
	RX1 day	0.23	*0.40*	*0.52*	*0.50*	*0.63*	*0.61*	*0.66*	*0.74*	*0.33*	*0.27*	*0.27*	−0.02
	RX5 day	0.25	*0.50*	*0.62*	*0.68*	*0.71*	*0.68*	*0.80*	*0.73*	*0.43*	*0.50*	*0.39*	0.23
FX5 day	PRCPprb	0.21	*0.44*	*0.35*	*0.41*	*0.59*	*0.52*	*0.51*	*0.58*	*0.42*	*0.30*	*0.32*	−0.07
	PRCPtot	*0.30*	*0.53*	*0.54*	*0.59*	*0.63*	*0.62*	*0.74*	*0.69*	*0.38*	*0.42*	*0.29*	0.02
	RX1 day	0.26	*0.38*	*0.51*	*0.48*	*0.60*	*0.56*	*0.67*	*0.76*	*0.35*	*0.28*	*0.28*	−0.03
	RX5 day	*0.29*	*0.48*	*0.63*	*0.67*	*0.68*	*0.63*	*0.80*	*0.75*	*0.46*	*0.52*	*0.39*	0.23

Italic denotes the correlations are statistically significant [α = 0.05] and the rest non-significant

**Fig. 3:**
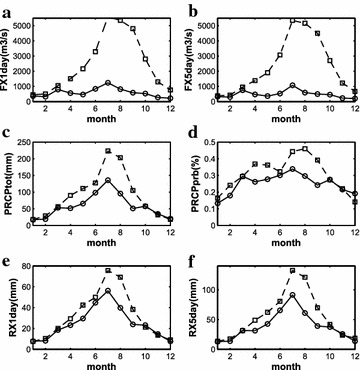
Seasonal variation of the streamflow and precipitation indices in the high-streamflow years (*dashed line*) and low-streamflow years (*solid line*). **a** FX1day; **b** FX5day; **c** PRCPtot; **d** PRCPprb; **e** RX1day; **f** RX5day

In this study, the high- and low-streamflow years are defined by standard deviation. When the streamflow volume of the year is one standard deviation above (below) the multiyear mean, this year is defined as high (low) streamflow year. According to this definition, nine high-streamflow years and nine low-streamflow years are identified in the study period 1958–2009. The values of precipitation indices and streamflow indices in the high- and low-streamflow years are presented in Table 7. The mean streamflow values during the entire study period are also included in Table 7 as references. From Table 7, it can be seen that the precipitation indices in the high-streamflow years are about 20–100 % greater than those in the low-streamflow years; however, the corresponding differences for the streamflow indices are even much greater, ranging from 350 to 570 %. This implies that when precipitation exceeds a certain “breaking point” or threshold, heavy rainfall-related flooding risks in the Huaihe River Basin will dramatically increase. The seasonal variations of the precipitation indices in high- and low-streamflow years are shown in Fig. 3. The results clearly indicate that in summer season all precipitation indices in the high-streamflow years are much greater than those in the low-streamflow years; however, in winter season the differences of indices between the high- and low-streamflow years are very small.

**Table 7:** Streamflow and precipitation indices in the high-/low-streamflow years and the entire period 1958–2009

Index	PRCPprb	PRCPtot	R95p	Rx1 day	RX5 day	SDII	FLow	FX1 day	FX5 day
High-streamflow years	0.31	1057.72	346.76	108.37	182.24	13.24	1569.62	6728.89	6478.89
Low-streamflow years	0.25	687.26	167.30	82.11	120.05	11.03	235.72	1674.33	1447.47
All years	0.28	886.00	257.25	93.83	147.16	12.31	818.39	4136.71	3920.22

**Fig. 4:**
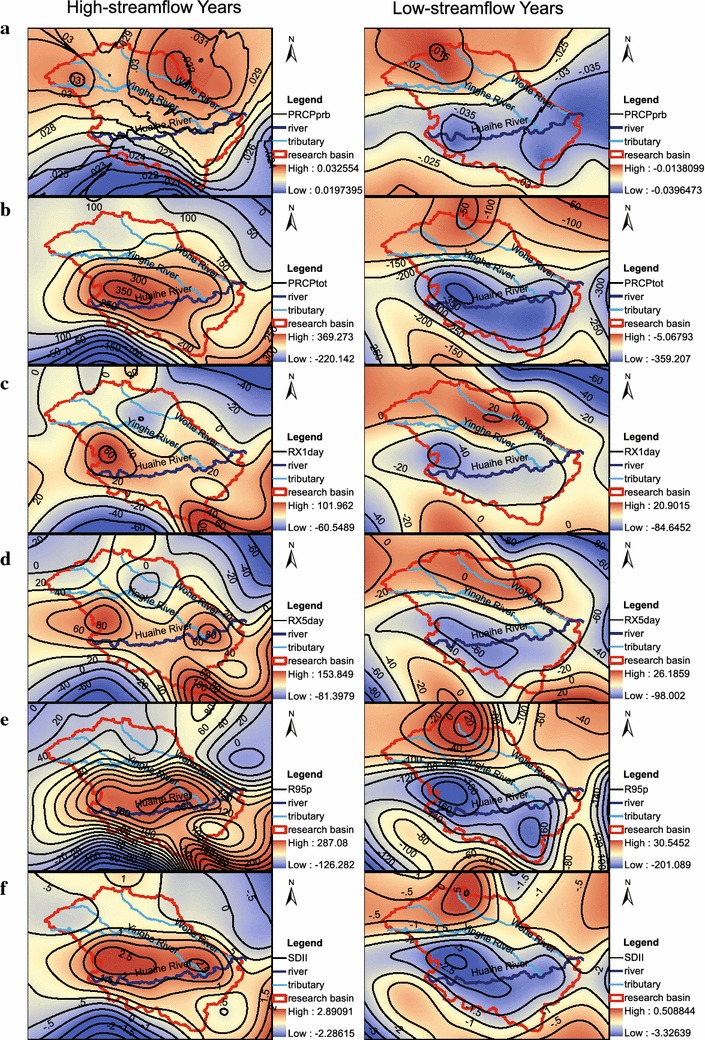
Spatial patterns of the precipitation index anomalies in the high- and low-streamflow years. **a** PRCPprb; **b **PRCPtot; **c** RX1day; **d** RX5day; **e** R95p; **f** SDII

The spatial patterns of the precipitation index anomalies in high- and low- streamflow years were examined. As shown in Fig. 4, the patterns of all precipitation index anomalies in the high-streamflow years are much different from those in the low-streamflow years. The region (especially in the upper and central Huaihe River Basin) with large positive index anomalies in the high-streamflow years will change to the region with large negative anomalies in the low- streamflow years. Consequently, this great inter-annual variation of precipitation anomaly causes the frequent floods and droughts in the upper and central basin.

**Fig. 5:**
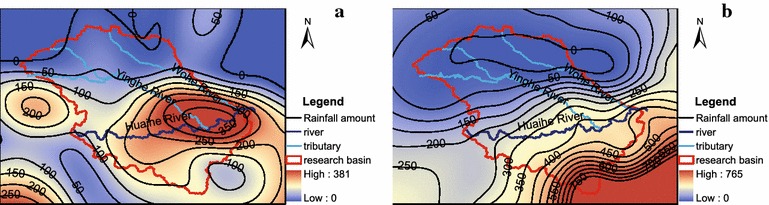
Spatial distributions of the total rainfall amount of two rainstorm events in 1991. **a** Rainstorm during June 10 to June 14;** b** rainstorm during June 29–July 11

**Fig. 6:**
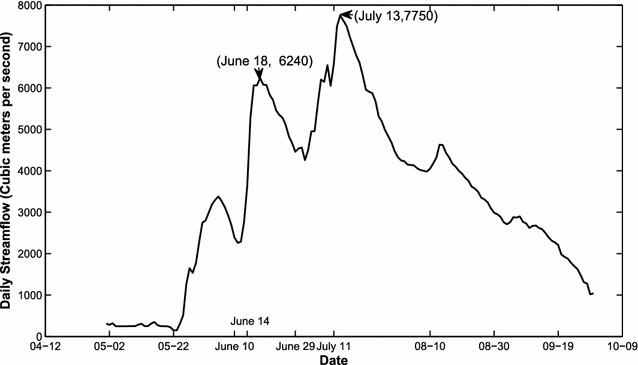
Daily discharge hydrograph during the flooding events in 1991 over the Huaihe River Basin

In June and July 1991, the Huaihe River Basin experienced the all basin severe floods. Specifically, there were two heavy rainfall storms occurred in June and July 1991. The first rainstorm lasted 5 days, from June 10 to June 14, the maximum total precipitation amount reached 381 mm. As shown in Fig. 5, the rainstorm covered a large area (more than ten meteorological stations) and its center located near Bengbu. As a result, streamflow volumes in the hydrological station Bengbu sharply increased from 2260 to 6240 (m^3^ s^−1^) in seven days (Fig. 6). The second rainstorm lasted 13 days from June 29 to July 11 with the maximum total precipitation of 765 mm in the southern basin. As a result, streamflow volumes in the hydrological station Bengbu dramatically increased from 4260 to 7750 (m^3^ s^−1^) from June 29 to July 11, which is the maximum streamflow level in 1991(Fig. 6). Annual precipitation and streamflow indices in the year of 1991 and the monthly values in the rainy season are listed in Table 8. Comparing Table 8 with Table 7, we can find that the streamflow and precipitation indices in 1991 are generally greater than those in the high-streamflow years.

**Table 8:** Streamflow and precipitation indices in 1991 vs averages of the entire period 1958–2009

Index		May	Jun	Jul	Aug	Sep	Annual
PRCPprb	1991	0.46	0.43	0.36	0.31	0.28	0.29
	All years	0.32	0.32	0.41	0.36	0.32	0.28
PRCPtot	1991	138.77	209.82	246.62	141.94	78.00	1090.24
	All years	88.95	119.33	189.52	135.92	84.75	886.00
RX1 day	1991	43.82	71.21	82.39	64.26	35.51	108.77
	All years	36.08	48.00	66.58	51.95	34.90	93.83
RX5 day	1991	71.67	130.96	165.30	105.44	53.64	206.00
	All years	54.40	73.51	112.11	84.18	59.39	147.16
FLow	1991	582.77	4268.67	5867.10	3900.32	2238.33	1685.03
	All years	649.10	728.38	2164.67	1896.20	1311.55	818.39
FX1 day	1991	2750.00	6240.00	7750.00	4630.00	2900.00	7750.00
	All years	1268.38	1763.99	3676.37	3021.29	2224.87	4136.71
FX5 day	1991	2002.00	6102.00	7516.00	4464.00	2836.00	7516.00
	All years	1111.84	1508.88	3468.44	2783.01	2042.34	3920.22

**Fig. 7:**
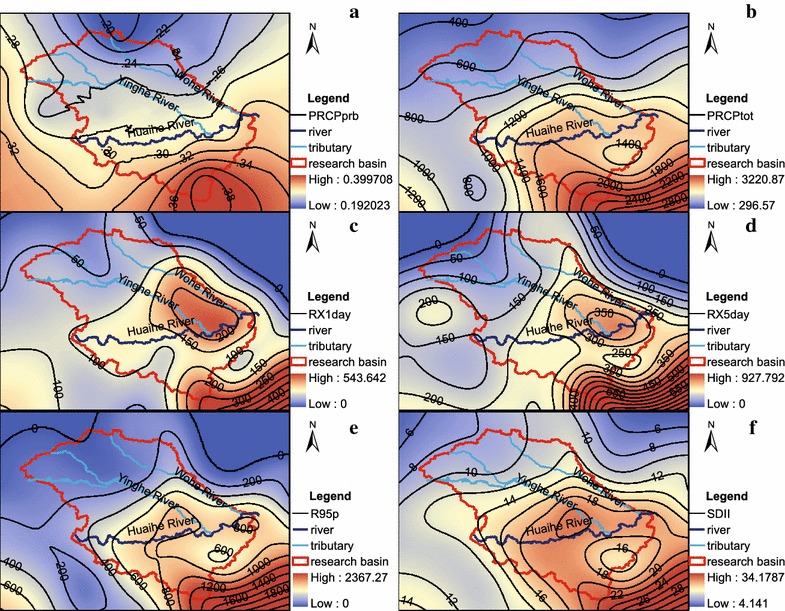
Spatial patterns of the precipitation indices in 1991. **a** PRCPprb; **b** PRCPtot; **c** RX1day; **d** RX5day; **e** R95p; **f **SDII

The spatial distributions of the precipitation indices in 1991 are shown in Fig. 7. In these precipitation indices, only the spatial pattern of PRCPprb (Fig. 7a) is roughly similar to that in the high-streamflow years (Fig. 4a), with high and low PRCPprb values in the southern basin and northern basin, respectively. In 1991, the spatial patterns of the precipitation intensity indices, RX1 day, RX5 day, R95p and SDII showed the combined influence of two heavy rainstorms, with high value center near Bengbu and high value area covered the southern basin (Fig. 7c–e). This case study indicated that the substantial increases in precipitation and torrential rainfall triggered the severe flooding over the Huaihe River Basin in June and July 1991.

## Conclusions

Based on the precipitation extremes indices defined by ETCCDMI, the hydrological impacts of precipitation extremes were studied. The conclusions from this study are as follows.

First, the seasonal contrast of the precipitation between the summer and autumn in the Huaihe River Basin, China, became more significant in the later years during the period 1958–2009. This result implies that the Huaihe River Basin has the top risks from heavy rainfall-related flooding in summer and more severe droughts in autumn.

Second, the extreme precipitation events or consecutive heavy rain day events resulted in the substantial increases in streamflow extremes, which was the main cause of the severe floods in the Huaihe River Basin. Although the seasonal variation of streamflow is greatly influenced by the human activities, the streamflow indices usually have a very strong connection with the precipitation indices at the annual scale. The heavy rainfall events highly impact on the streamflow extremes in the Huaihe River Basin.

Finally, the spatial patterns of precipitation anomalies have great impact on streamflow in the Huaihe River Basin. The large precipitation anomalies in the upper and central basin near the Huaihe mainstream are the major causes of the area frequent floods and droughts.

## References

[CR1] Aguilar E, Peterson TC, Obando PR (2005). Changes in precipitation and temperature extremes in Central America and northern South America. J Geophys Res.

[CR2] Alexander LV, Zhang X, Peterson TC (2006). Global observational changes in daily climate extremes of temperature and precipitation. J Geophys.

[CR3] Allan RP, Soden BJ (2008). Atmospheric warming and the amplification of precipitation extremes. Science.

[CR4] Bartholy J, Pongrácz R (2007). Regional analysis of extreme temperature and precipitation indices for the Carpathian Basin from 1946 to 2001. Glob Planet Change.

[CR5] Bartholy J, Pongrácz R (2010). Analysis of precipitation conditions for the Carpathian Basin based on extreme indices in the 20th century and climate simulations for 2050 and 2100. Phys Chem Earth.

[CR6] Bocheva L, Marinova T, Simeonov P (2009). Variability and trends of extreme precipitation events over Bulgaria (1961–2005). Atmos Res.

[CR7] Brown PJ, Bradley RS, Keimig FT (2010). Changes in extreme climate indices for the Northeastern United States, 1870–2005. J Clim.

[CR8] Cheng CS, Li G, Li Q, Auld H (2010). A synoptic weather typing approach to simulate daily rainfall and extremes in Ontario, Canada: potential for climate change projections. J Appl Meteorol Climatol.

[CR9] Cheng CS, Li G, Li Q, Auld H (2011). A synoptic weather-typing approach to project future daily rainfall and extremes at local scale in Ontario, Canada. J Clim.

[CR10] Choi G, Collins D, Ren GY (2009). Changes in means and extreme events of temperature and precipitation in the Asia-Pacific network region, 1955–2007. Int J Climatol.

[CR11] Chu PS, Chen YR, Schroeder TA (2010). Changes in precipitation extremes in the Hawaiian Islands in a warming climate. J Clim.

[CR12] Ding YH (2008). Introduction to China’s climate change science (in Chinese).

[CR13] Dong Q, Chen X, Chen TX (2011). Characteristics and changes of extreme precipitation in the Yellow-Huaihe and Yangtze-Huaihe Rivers Basins, China. J Clim.

[CR14] Easterling DR, Meehl GA, Parmesan C (2000). Climate extremes: observations, modeling, and impacts. Science.

[CR15] Gemmer M, Fischer T, Jiang T (2011). Trends in precipitation extremes in the Zhujiang River Basin, South China. J Clim.

[CR16] Groisman PY, Karl TR, Easterling DR (1999). Changes in the probability of heavy precipitation: important indicators of climatic change. Clim Change.

[CR17] Groisman PY, Knight RW, Easterling DR (2005). Trends in intense precipitation in the climate record. J Clim.

[CR18] Hundecha Y, Bárdossy A (2005). Trends in daily precipitation and temperature extremes across western Germany in the second half of the 20th century. Int J Climatol.

[CR19] IPCC (2001). Climate change. The science of climate change. Contribution of working group I to the third assessment report of the intergovernmental panel on climate change [R].

[CR20] IPCC (2007). Climate change The physical science basis. Contribution of working group I to the fourth assessment report of the intergovernmental panel on climate change [R].

[CR21] IPCC (2013). Climate change. The physical science basis. Contribution of working group I to the fourth assessment report of the intergovernmental panel on climate change [R].

[CR22] Jiang DJ, Wang K, Li Z (2011). Variability of extreme summer precipitation over Circum-Bohai-Sea region during 1961–2008. Theor Appl Climatol..

[CR23] Karl TR, Knight RW (1998). Secular trends of precipitation amount, frequency, and intensity in the USA. Bull Am Meteorol Soc.

[CR24] Kendall MG (1975). Rank correlation measures.

[CR25] Kioutsioukis I, Melas D, Zerefos C (2010). Statistical assessment of changes in climate extremes over Greece (1955–2002). Int J Climatol.

[CR26] Kostopoulou E, Jones PD (2005). Assessment of climate extremes in the Eastern Mediterranean. Meteorol Atmos Phys.

[CR27] Kruger AC (2006). Observed trends in daily precipitation indices in South Africa: 1910–2004. Int J Climatol.

[CR28] Kunkel KE, Andsager K, Easterling DR (1999). Long-term trends in extreme precipitation events over the conterminous United States and Canada. J Clim.

[CR29] Łupikasza EB, H¨ansel S, Matschullat J (2011). Regional and seasonal variability of extreme precipitation trends in southern Poland and central-eastern Germany 1951–2006. Int J Climatol.

[CR30] Mann HB (1945). Non-parametric tests against trend. Econometric.

[CR31] Manton MJ, Della-Marta PM, Haylock MR (2001). Trends in extreme daily rainfall and temperature in Southeast Asia and the South Pacific. Int J Climatol.

[CR32] Meehl GA, Karl TR, Easterling DR (2000). An introduction to trends in extreme weather and climate events: observations, socioeconomic impacts, terrestrial ecological impacts, and model projections. Bull Am Meteorol Soc.

[CR33] Michael LG, Bradley RS (2007). Variations of twentieth-century temperature and precipitation extreme indicators in the Northeast United States. J Clim.

[CR34] Mishra AK, Singh VP (2010). Changes in extreme precipitation in Texas. J Geophys Res.

[CR35] Moberg A, Jones PD (2005). Trends in indices for extremes in daily temperature and precipitation in central and Western Europe, 1901–99. Int J Climatol.

[CR36] Nicholls N, Murray W (1999). Workshop on indices and indicators for climate extremes: precipitation. Clim Change.

[CR37] Pal I, Al-Tabbaa A (2009). Trends in seasonal precipitation extremes—an indicator of ‘climate change’ in Kerala, India. J Hydrol.

[CR38] Ren GY (2007). Climatic change and china’s water resources.

[CR39] Ren K (2011). Review and reflection of the reservoir flood dispatching in Huaihe River Basin. Zhihuai.

[CR40] Roy SS, Balling RC (2004). Trends in extreme daily precipitation indices in India. Int J Climatol.

[CR41] Roy SS, Balling RC (2009). Evaluation of extreme precipitation indices using daily records (1910–2000) from India. Weather.

[CR42] Santos CAC, Neale MU, Rao TV (2011). Trends in indices for extremes in daily temperature and precipitation over Utah, USA. Int J Climatol.

[CR43] Sarahí AR, Cavazos T (2010). Regional trends of daily precipitation indices in northwest Mexico and southwest United States. J Geophys Res.

[CR44] Sen PK (1968). Estimates of the regression coefficient based on Kendall’s tau. J Am Stat Assoc.

[CR45] Su BD, Jiang T, Ren GY (2006). Observational trends of precipitation extremes in the Yangtze River Basin during 1960 to 2004. Adv Clim Change Res.

[CR46] Tank AMG, Können GP (2003). Trends in indices of daily temperature and precipitation extremes in Europe, 1946–1999. J Clim.

[CR47] Vincent LA, Aguilar E, Saindou M (2011). Observed trends in indices of daily and extreme temperature and precipitation for the countries of the western Indian Ocean, 1961–2008. J Geophys Res.

[CR48] Wang MH, Xie Q, Wang HY (2003). Impact of future climate change on runoff depth of the Huaihe drainage basin. Geogr Res.

[CR49] Wang W, Chen X, Shi P, Van Gelder PHAJM (2008). Detecting changes in extreme precipitation and extreme streamflow in the Dongjiang River Basin in southern China. Hydrol Earth Syst Sci Discuss.

[CR50] Xu L, Ou ZZ (2012). Agricultural water problems and protection countermeasure analysis in Huaihe River Basin. Water Resour Dev Res.

[CR51] You QL, Kang SC, Aguilar E (2008). Changes in daily climate extremes in the eastern and central Tibetan Plateau during 1961–2005. J Geophys Res.

[CR52] Zhai PM, Sun AJ, Ren FM (1999). Changes of climate extremes in China. Clim Change.

[CR53] Zhai PM, Zhang XB, Wan H (2005). Trends in total precipitation and frequency of daily precipitation extremes over China. J Clim.

[CR54] Zhan MJ, Yin JM, Zhang YZ (2011). Analysis on characteristic of precipitation in Poyang Lake basin from 1959 to 2008. Proced Environ Sci.

[CR55] Zhang ZX, Zhang Q, Jiang T (2007). Changing features of extreme precipitation in the Yangtze River basin during 1961–2002. J Geogr Sci.

[CR56] Zhang AY, Gao X, Ren GY (2008). Characteristic of extreme precipitation events change in Central North China in recent 45 years. Arid Meteorol.

[CR57] Zhang JL, Wang J, Gan QH (2009). Temporal and spatial variation characteristics of extreme precipitation events in the Yangtze and Huaihe River Basin of China from 1961 to 2006. J Anhui Agri Sci..

